# Acute Changes in Liver and Spleen Stiffness Following Endoscopic Variceal Ligation in Advanced Liver Disease—A Pilot Study

**DOI:** 10.3390/jcm15020816

**Published:** 2026-01-20

**Authors:** Esra Görgülü, Eva Herrmann, Jonel Trebicka, Alexander Queck, Georg Dultz, Vitali Koch, Stefan Zeuzem, Jörg Bojunga, Viola Knop, Florian Alexander Michael, Mireen Friedrich Rust

**Affiliations:** 1 Medical Clinic 1, University Hospital Frankfurt, Goethe University Frankfurt, Theodor-Stern-Kai 7, 60590 Frankfurt am Main, Germany; 2 Institute of Biostatistics and Mathematical Modelling, Faculty of Medicine, Goethe University Frankfurt, Theodor-Stern-Kai 7, 60590 Frankfurt am Main, Germany; 3 Department of Gastroenterology and Hepatology, University Hospital Münster, Albert-Schweitzer-Straße 33, 48149 Münster, Germany; 4 Department of Diagnostic and Interventional Radiology, University Hospital Frankfurt, Goethe University Frankfurt, Theodor-Stern-Kai 7, 60590 Frankfurt am Main, Germany

**Keywords:** transient elastography, esophageal varices, spleen stiffness measurement, endoscopic variceal ligation, liver cirrhosis

## Abstract

**Background/Objectives:** Endoscopic variceal ligation (EVL) is a common treatment for preventing variceal bleeding in patients with advanced chronic liver disease (ACLD). However, its acute hemodynamic impact is typically assessed using invasive methods, and there is data on short-term spleen stiffness (SS) dynamics are limited. This pilot study aimed to quantify short-interval changes in liver stiffness (LS) and SS following EVL using transient elastography (TE), and to explore their associations with clinical and laboratory parameters. **Methods:** This prospective observational study enrolled adults with advanced liver disease undergoing esophagogastroduodenoscopy (EGD) with or without EVL at a tertiary center. Liver and spleen TE were performed in a fasted state immediately before endoscopy and repeated within 12 h after EVL. Organ-specific probes and predefined quality criteria were used, and non-parametric methods were applied to assess within-patient changes and correlations. **Results:** Fifty patients were included in the study: 21 underwent EVL, while the remaining 29 underwent diagnostic endoscopies only. The most common cause was alcohol-related liver disease. Within the EVL subgroup, the median liver stiffness (LSM) increased from 27.6 kPa to 45.1 kPa, and the median spleen stiffness (SSM) increased from 59.9 kPa to 98.3 kPa, both within 12 h. While these increases showed a uniform direction, they did not reach statistical significance. A higher baseline SS predicted a greater LS increase, and stiffness measures correlated with creatinine, disease duration, Child–Pugh class, albumin and ascites. **Conclusions:** Short-term increases in liver and spleen stiffness following EVL are consistent with acute hemodynamic alterations, such as increased hepatic perfusion and splenic congestion, rather than structural remodeling. These findings, beyond changes in stiffness alone, support the feasibility of integrating TE, particularly the measurement of SS, into early peri-procedural hemodynamic surveillance after EVL. They also justify larger studies with serial time points and direct portal pressure validation.

## 1. Introduction

Liver cirrhosis is the final stage of chronic liver disease and is greatly affected by the presence and severity of portal hypertension (PH) [1]. PH is not a static condition but a dynamic hemodynamic disorder resulting from increased intrahepatic resistance and altered splanchnic blood flow [2]. Together these factors drive the development of clinically relevant complications. Progression from compensated to decompensated cirrhosis reflects structural liver damage and evolving portal and splanchnic hemodynamics. This underlines the need for tools that can capture these changes over time [3,4]. Evaluating esophageal varices (EV) is particularly important in patients with decompensated cirrhosis, as this is essential for the risk stratification and clinical management [5].

Although hepatic venous pressure gradient (HVPG) measurements and esophagogastroduodenoscopy (EGD) are considered the reference standards for diagnosing clinically significant portal hypertension (CSPH) and assessing esophageal varices, both methods are invasive and resource-intensive. They are also poorly suited to repeated or short-interval monitoring [5]. Therefore, in recent years, non-invasive approaches have recently gained importance, in order to improve risk stratification while reducing procedural burden.

Liver stiffness measurement (LSM) by transient elastography (TE) has emerged as a valuable surrogate marker of PH [6,7,8,9,10]. However, its diagnostic accuracy decreases at higher HVPG levels (>10 mmHg), where stiffness values tend to plateau and become less reflective of dynamic hemodynamic changes [11]. These limitations have prompted the development of additional non-invasive markers to provide a more accurate picture of portal and splanchnic hemodynamics [12,13,14,15,16,17,18,19,20].

In response to these limitations, recent consensus statements have increasingly emphasized the role of spleen stiffness measurement (SSM) in non-invasively assessing PH. The Baveno VII criteria incorporate SSM into risk stratification algorithms for clinically significant PH and high-risk varices, recognizing that SSM is more closely related to portal and splanchnic hemodynamics than LS alone [21].

Unlike LS, which predominantly reflects intrahepatic resistance and fibrosis, SS directly reflects splenic congestion, hyperplasia, and changes in portal inflow. This makes it a sensitive marker of advanced PH and dynamic hemodynamic alterations [22]. Several studies have demonstrated that SSM exhibits a stronger and more linear association with portal pressure at higher HVPG levels, whereas LS tends to plateau [23].

Building on this concept, integrative non-invasive models that combine SS with laboratory parameters have recently been proposed to further refine risk stratification and reduce the diagnostic ‘grey zone’ inherent to single-parameter approaches [24]. Scores such as the VITRO score exemplify this strategy, emphasizing the evolving role of SS as a hemodynamic biomarker rather than merely an adjunct to LSM [25].

Measurement of spleen stiffness (SSM) has been shown to be highly valuable in identifying patients with high-risk varices and in predicting liver-related outcomes across the different stages of PH [26,27,28]. These findings support the use of SSM as a robust, non-invasive marker of PH severity, and justify its integration into contemporary risk stratification strategies.

Current guidelines recommend using non-selective beta-blockers to prevent decompensation in patients with clinically significant PH. For compensated patients with high-risk varices who have contraindications or are intolerant to pharmacological therapy, endoscopic variceal ligation (EVL) is the standard of care for primary bleeding prophylaxis [21]. Beyond its well-established role in preventing bleeding, EVL is an acute hemodynamic intervention that alters portal venous flow and pressure distribution. Previous studies have demonstrated that rapid shifts in portal pressure following EVL can immediately alter LS, highlighting the procedure’s dynamic hemodynamic impact [29]. Furthermore, well-established predictors of early post-ligation bleeding, including low hemoglobin levels, high MELD scores, the presence of gastric varices, and procedural factors, emphasize the complexity of the early post-interventional phase [30,31].

However, to date, no studies have yet systematically investigated the acute effects of EVL on SS. Given the central role of splenic congestion and splanchnic hemodynamics in PH, this represents a significant knowledge gap. The aim of this pilot study was therefore to assess short-term changes in liver and spleen stiffness before and after EVL in patients with advanced liver disease using TE. By focusing on the immediate post-procedural period, the study aimed to explore the feasibility of using SSM as an early, non-invasive hemodynamic surveillance tool following EVL, and to inform future post-interventional monitoring strategies in PH.

## 2. Materials and Methods

### 2.1. Study Design and Objective

This prospective, observational, pilot study aimed to quantify the short-term changes in liver stiffness (LSM) and spleen stiffness (SSM) before and after EVL in patients with advanced liver disease. These changes were measured using transient elastography (TE; FibroScan Expert 630, Echosens, Paris, France). The primary endpoint of the study was the change in LSM and SSM within patients from pre- to post-EVL. The short post-interventional assessment window was chosen in order to capture acute hemodynamic changes rather than structural remodeling processes. As a pilot study, the design aimed to generate hypothesis-forming data on the feasibility and variability of early changes in liver and spleen stiffness following EVL.

### 2.2. Participants and Setting

Consecutive adults (aged 18 years or over) with advanced liver disease or cirrhosis, who were scheduled to undergo an EGD with possible EVL at Frankfurt University Hospital, were screened. Inclusion criteria for participation in the study entailed ongoing EVL therapy or an initial ligation evaluation. Patients were required to provide written informed consent to participate in the study. The endoscopists were unaware of the study participants’ status and were fully blinded to all TE results. The clinical decision to perform EVL was made entirely independently of the study. The following criteria were used to determine exclusion from the study:

- decompensated cirrhosis;
- American Society of Anesthesiologists (ASA) physical status ≥ IV;
- legal incapacity without a guardian;
- Current pregnancy.

For the purposes of patient selection, decompensated cirrhosis was defined as the presence of acute or unstable decompensation requiring immediate therapeutic intervention. This included uncontrolled or refractory ascites, active gastrointestinal bleeding, acute hepatic encephalopathy, severe infection, or acute kidney injury. Patients with a history of prior decompensation who were clinically stable (e.g., controlled ascites or previous episodes of hepatic encephalopathy under ongoing treatment) were eligible for inclusion. Written informed consent was obtained from all participants. The study was approved by the Ethics Committee of Goethe University Frankfurt (No. 20-653, 11 August 2020).

### 2.3. Transient Elastography Protocol

TE was performed on a FibroScan Expert 630 (Echosens, Paris, France) using the validated 50-Hz liver probe and the 100-Hz spleen-specific probe for liver and spleen stiffness, respectively, in accordance with the manufacturer’s guidelines and the established best practice recommendations. Patients were examined in a fasted state and an additional curved ultrasound probe was used to optimize organ localization and the acoustic window, particularly for spleen positioning. All TE examinations were performed by trained operators with documented experience in liver and spleen stiffness measurement. Measurements were considered reliable if at least 10 valid acquisitions were obtained, with the median value expressed in kilopascals (kPa). The interquartile range-to-median ratio was also considered if it was within the accepted quality thresholds, in accordance with current recommendations. Patients were examined in the supine position with their right arm maximally abducted for LSM, while SSM were performed in the left lateral decubitus position to optimize the acoustic window.

XL probes were used when necessary, as indicated by the patient’s body habitus, and their use was meticulously documented as a potential confounding factor. To enhance the robustness of SSM, it is recommended that a valid series includes a minimum of ten measurements. Where feasible, at least twelve acquisitions should be made, given the higher variability within examinations at 100 Hz.

TE was performed prior to EGD and, for those undergoing EVL, was repeated within 12 h following ligation. The 12-h interval after EVL was selected for the post-EVL measurement to reflect early post-procedural hemodynamic changes while minimizing the impact of delayed inflammatory or clinical events.

### 2.4. Endoscopic Ligation and Variceal Grading

All patients received the same pre-procedural information and were sedated with propofol in accordance with the institutional guidelines. EVL was indicated for patients with high-risk varices, categorized according to the Paquet grading system [15]. Specifically, grade 2 with red signs and grades 3–4 were designated as high-risk varices. EVL was evaluated in accordance with contemporary consensus guidelines. The endoscopic team adhered to standard institutional protocols during the procedure. Patients and endoscopists were blinded to the TE results, and study participation did not alter standard care. EVL was performed using standard multiband ligation devices according to institutional practice. The number of bands applied was at the endoscopist’s discretion and based on the size and morphology of the varices. This information was documented for exploratory analyses.

### 2.5. Data Collection and Secondary Variables

A comprehensive dataset was compiled, including demographics, clinical findings, the underlying etiology, and medication regimens, including anticoagulation and non-selective beta-blockers. Laboratory parameters were obtained from routine clinical testing performed within 24 h of the endoscopic procedure, ensuring temporal proximity to elastography measurements. The presence of a transjugular intrahepatic portosystemic shunt (TIPS) was also documented, alongside endoscopic details such as the number of bands applied. If data were available from routine care, invasive hemodynamic data and Model for End-Stage Liver Disease (MELD) scores were to be extracted at predefined time points. Documented potential confounders included probe type (M vs. XL), deviations in fasting status, and measurement quality indices.

### 2.6. Statistical Analysis

The analyses were performed using SPSS version 29.0 and GraphPad Prism version 9.5. Descriptive statistics included means, standard deviations, medians, interquartile ranges, and frequencies, as appropriate. Within-patient pre/post changes were analyzed using Wilcoxon matched-pairs signed-rank tests. Between-group comparisons were performed using the Mann–Whitney U or Kruskal–Wallis tests, and Spearman’s rank correlation was used to investigate associations. The significance level was set at *p* < 0.05 (two-sided). An outlier that was identified during non-parametric testing was reviewed. Subsequent chart review revealed the presence of hepatocellular carcinoma (HCC), which plausibly explains the aberrant stiffness values. This case was excluded from efficacy analyses but was retained for sensitivity checks where applicable. The graphical abstract was created using BioRender under an institutional license. Supplementary Figures were generated in R version 4.4.2 using the MetaboAnalystR 4.0 package.

## 3. Results

### 3.1. Patient Characteristics

**Figure 1:**
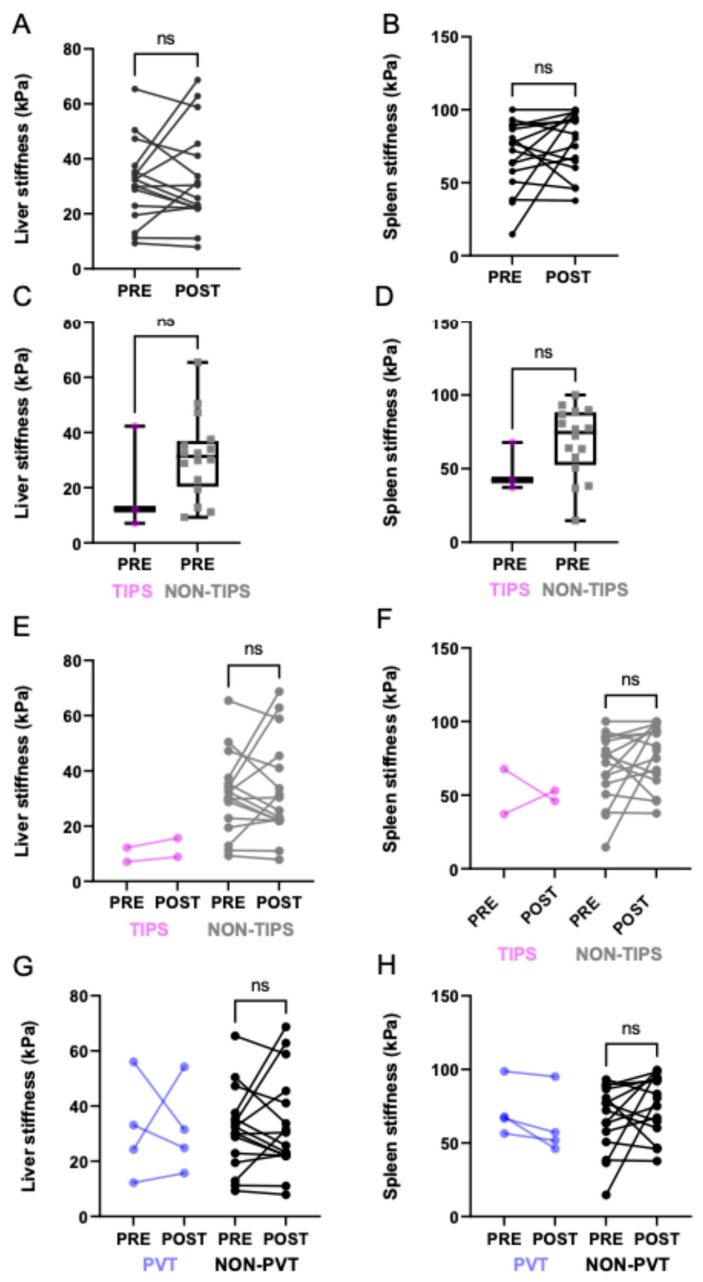
Distribution of liver and spleen stiffness values before and after ligation therapy. Light purple symbols represent patients with TIPS, dark purple symbols represent patients with portal vein thrombosis (PVT), and black symbols indicate patients without either condition. Lines connect paired pre- and post-ligation measurements. Boxplots represent the median and interquartile range. ns indicates non-significant differences. (**A**) Liver stiffness before and after EVL. (**B**) Spleen stiffness before and after EVL. (**C**) Liver stiffness before EVL in TIPS versus non-TIPS patients. (**D**) Spleen stiffness before EVL in TIPS versus non-TIPS patients. (**E**) Changes in liver stiffness pre- and post-EVL, stratified by TIPS status. (**F**) Changes in spleen stiffness before and after EVL, stratified by TIPS status. (**G**) Changes in liver stiffness pre- and post-EVL, stratified by portal vein thrombosis. (**H**) Changes in spleen stiffness pre- and post-EVL, stratified by portal vein thrombosis.

Between April 2021 and January 2023, 54 patients with advanced liver disease underwent screening. One patient declined to participate, and three did not meet the inclusion criteria. This left 50 patients for analysis: 21 underwent EVL and 29 underwent diagnostic EGD procedures only (see Figure 1). The most prevalent etiology was alcohol-related liver disease, followed by viral, autoimmune, and metabolic-associated steatohepatitis. The baseline demographic and clinical characteristics of the study participants are outlined in Table 1.

Comparing patients who underwent EVL with those who did not revealed a higher prevalence of ascites (81% vs. 62%, *p* < 0.001) and lower platelet counts (99 ± 43 vs. 154 ± 112 × 10^9^/L, *p* < 0.05) in the former group. However, the two groups were comparable with respect to age, sex distribution, MELD score, Child–Pugh class, and creatinine levels. All EVL procedures targeted high-risk varices. Of these EVL procedures, 57% were performed for primary prophylaxis and 43% for secondary prophylaxis.

The cohort reflects a real-world population of patients with advanced liver disease who undergo endoscopic assessment as part of routine clinical care at a tertiary center. The variables reported in Table 1 include both findings at the time of study inclusion and a history of PH-related complications recorded from the patient’s previous medical history. Patients undergoing EVL had a more frequent documented history of ascites and showed lower platelet counts. Age, sex distribution, MELD score, Child–Pugh score, and serum creatinine levels were comparable between the two groups (see Table 1).

Baseline demographic and disease characteristics:

### 3.2. Changes in Liver and Spleen Stiffness Following EVL

**Figure 2:**
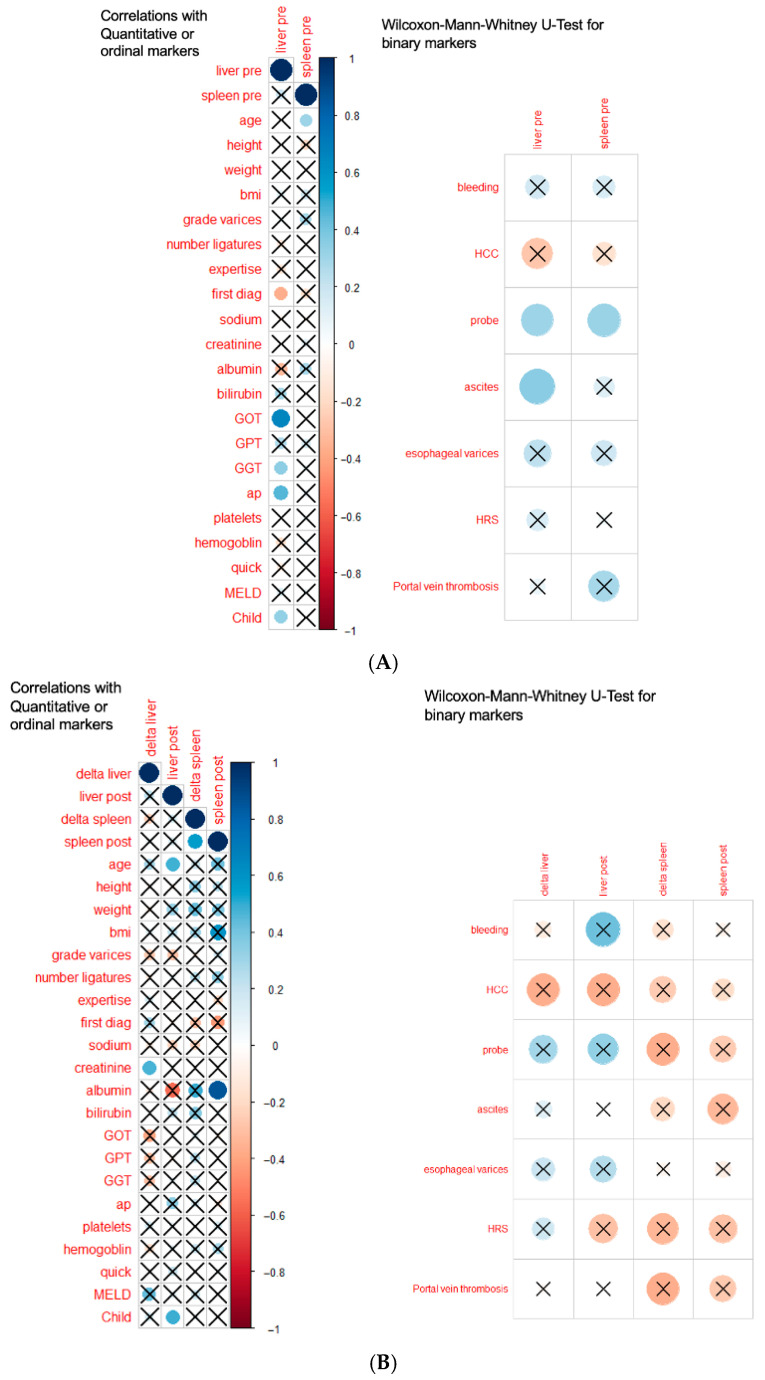
(**A**) Correlations between pre-procedural liver (LS) and spleen (SS) stiffness and quantitative and ordinal clinical and laboratory markers in all patients (*n* = 50), as well as group comparisons for binary variables. (**B**) Correlations of changes in liver and spleen stiffness (ΔLS and ΔSS) and post-procedural values with clinical parameters in patients undergoing endoscopic variceal ligation (EVL; *n* = 21). **Explanation of symbols:** The color of the circle indicates the direction and strength of the association (blue = positive correlation/association; red = negative correlation/association), with color intensity proportional to the correlation coefficient. Circle size reflects the magnitude of the effect. An ‘×’ indicates non-significant results (*p* ≥ 0.05). Spearman’s rank correlation was used for quantitative and ordinal variables, and the Wilcoxon–Mann–Whitney U test was applied for binary variables. All *p*-values are two-sided.

TE performed within 12 h of EVL revealed uniform directional increases in stiffness in both the liver and the spleen. In the EVL subgroup (*n* = 21), the median LS increased from 27.6 kPa (range 16.0–43.5 kPa) to 45.1 kPa (range 19.2–63.4 kPa), corresponding to a median increase of +17.5 kPa. Meanwhile, the median SS increased from 59.9 kPa (range 37.4–96.5 kPa) to 98.3 kPa (range 45.0–139.7 kPa), corresponding to an increase of +38.4 kPa (see Figure 2A,B). Despite these consistent trends, changes in LSM and SSM did not reach statistical significance (*p* > 0.05), reflecting high inter-individual variability and the small sample size. At the individual patient level, most patients undergoing EVL exhibited an increase in both liver and spleen stiffness following the procedure, although the magnitude of change varied substantially between individuals. No consistent pattern of decrease was observed in the EVL group, highlighting the uniform directionality of the observed response despite considerable inter-individual variability.

At baseline, no statistically significant differences in liver or spleen stiffness were observed between the EVL and non-EVL groups (median overall LSM ≈30.0 kPa, U = 383.5, *p* = 0.16; median overall SSM ≈ 67.5 kPa, U = 303.0, *p* = 0.75). Pre-ligation measurements obtained with the XL probe yielded significantly higher liver and spleen stiffness values than those obtained with the standard probe (*p* = 0.0322 and *p* = 0.0302), and this trend remained consistent across probe types from pre- to post-EVL. Notably, although absolute stiffness values differed according to probe type, the direction of change from pre- to post-EVL remained consistent. This suggests that relative changes over time are preserved irrespective of technical factors.

### 3.3. Correlations and Subgroup Analyses

Correlation analyses revealed several moderate associations between stiffness parameters and clinical or laboratory variables. However, no strong correlations were observed across the dataset. Baseline LS was significantly correlated with serum creatinine levels (ρ = 0.41, *p* = 0.02) and disease duration (ρ = 0.37, *p* = 0.04), and a statistically significant correlation was observed (*p* = 0.02). Furthermore, higher Child–Pugh scores and elevated levels of alanine aminotransferase (ALT), gamma-glutamyl transferase (GGT), and alkaline phosphatase (ALP), as well as the presence of ascites, were found to be associated with higher baseline LS (*p* = 0.0152). In the EVL subgroup, higher Child–Pugh scores were associated with higher LS both before (r = 0.3364, *p* = 0.028) and after (r = 0.4937, *p* = 0.023) ligation. Post-EVL LS demonstrated a further correlation with age (ρ = 0.486, *p* = 0.0254).

The magnitude of the change in liver stiffness change (ΔLS) showed a positive correlation with baseline SS (ρ ≈ 0.47, *p* ≈ 0.03), indicating that patients with higher SSM at baseline experienced greater increases in LS after EVL. Additionally, a positive correlation was identified between the increase in LS after EVL and serum creatinine levels (ρ = 0.476, *p* = 0.0293). However, no significant associations were observed between stiffness changes and procedural variables, including the number of bands applied or the indication for EVL (primary versus secondary prophylaxis). Baseline SS showed an inverse correlation with serum albumin (ρ = −0.43, *p* = 0.01), with higher values observed in older patients. Following EVL, elevated SSM values were observed in patients with elevated albumin levels. No consistent correlation was detected between the Child–Pugh score and SSM (both *p* > 0.20).

Exploratory subgroup analyses revealed that patients with TIPS (*n* = 4) exhibited lower baseline liver and spleen stiffness, and tended to demonstrate decreases rather than increases following EVL. In contrast, patients with portal vein thrombosis (*n* = 8) demonstrated heterogeneous stiffness changes without a uniform pattern. Due to the small number of patients in these exploratory subgroups, no formal statistical testing was performed and these findings should be interpreted descriptively.

## 4. Discussion

This pilot study systematically evaluated short-interval changes in both liver and spleen stiffness following EVL using TE, extending prior work that primarily focused on hepatic stiffness or portal pressure alone [13]. While the role of SSM in risk stratification and screening for esophageal varices has been well established in previous studies, most existing data are derived from static, cross-sectional assessments [32]. In contrast, little is known about the short-term dynamic behavior of SS in response to therapeutic interventions that acutely modify portal hemodynamics. By focusing on immediate post-EVL changes, the present study builds upon previous research by addressing a peri-interventional time window that has so far remained largely unexplored. The data support the concept that elastography can capture peri-procedural hemodynamic shifts beyond static risk assessment, highlighting the complementary role of SS as an indicator of splanchnic congestion.

Although the observed increases in liver and spleen stiffness following EVL were not statistically significant the direction of these findings was consistent with previous research.

Piecha et al. [29] demonstrated an immediate rise in LS driven by acute changes in portal pressure, while Lo et al. [33] reported increases in portal pressure after ligation, particularly in the absence of compensatory collateral circulation. Taken together, these data support the interpretation that short-term stiffness elevations after EVL predominantly reflect transient hemodynamic perturbations rather than structural remodeling. The physiology of EVL involves occluding low-resistance portosystemic shunts, thereby enhancing intrahepatic perfusion and increasing sinusoidal pressure. However, interruption of variceal outflow may trigger venous congestion and splenic pooling, collectively augmenting liver and spleen stiffness.

In addition to acute changes in liver and spleen stiffness, EVL may also influence a broader range of manifestations related to PH by altering portal hemodynamics and redistributing flow. Previous studies have shown that EVL can lead to the development or progression of portal hypertensive gastropathy and portal hypertension-related polyps. This suggests that variceal obliteration may increase pressure and flow in the splanchnic circulation [34,35]. These phenomena have been attributed to the diversion of portal flow from low-resistance portosystemic collaterals towards the gastric and splenic vascular beds. In this context, the observed post-EVL increase in SS in the present study may reflect not only an acute local response as well as broader splanchnic congestion following ligation.

The growing global prevalence of liver cirrhosis and its complications highlights the clinical relevance of non-invasive monitoring strategies [36]. As patients progress to more advanced stages of the disease, they undergo an increasing number of diagnostic and therapeutic interventions, while simultaneously facing an elevated risk of complications during procedures due to PH, coagulopathy, and impaired organ function [37,38]. In this context, repeated invasive procedures such as HVPG measurements or endoscopy may be poorly tolerated or impractical, thereby reinforcing the need for reliable, non-invasive tools that can assess PH dynamics over time.

According to the Baveno VII criteria, patients with LS levels below 20 kPa and platelet counts above 150,000/µL can safely avoid screening endoscopy [21]. In this real-world cohort, however, only a minority met these low-risk criteria: specifically, three out of 51 patients had LS below 20 kPa and platelet counts above 150,000/µL. Most patients underwent EGD due to a history of bleeding, an overall high-risk profile, or ongoing EVL programs, rather than in accordance with Baveno VII’s strict recommendations. Rather than diminishing the significance of the current findings, this emphasizes that stiffness monitoring may be especially pertinent in higher-risk populations, where individualized surveillance strategies are necessary. Importantly, the findings of this study do not support the use of elastography in decision-making regarding EVL itself, but rather highlight its potential role in peri-procedural surveillance. Spleen stiffness measurement (SSM) may provide additional information on early post-interventional hemodynamic changes that could help to identify patients at risk of subsequent portal hypertension-related complications.

Beyond group comparisons, the relationship between TE values and laboratory parameters, disease duration and technical factors was explored. Baseline LS was found to be correlated with renal function, disease duration, and liver function markers. This finding is consistent with prior research establishing a link between fibrosis, inflammation, systemic factors, and stiffness readouts. As expected, SS exhibited hemodynamic behavior and demonstrated additional correlations with albumin, suggesting a potential connection with synthetic function and vascular compensation that requires further investigation. Exploratory subgroup analyses revealed that TIPS recipients exhibited lower baseline stiffness and post-EVL decreases, consistent with portal pressure unloading. In contrast, patients with portal vein thrombosis exhibited heterogeneous trajectories, plausibly reflecting variable collateralization and flow patterns. No consistent differences in stiffness changes were observed across ligation-related subgroups (e.g., number or timing of bands), and numerical trends did not reach statistical significance, likely due to the modest sample size.

During the study, it was found that technical aspects were relevant. Despite having been validated, the 100-Hz spleen displayed greater variability. Furthermore, using the XL probe resulted in higher absolute liver and spleen stiffness values while maintaining the direction of pre/post changes. These observations emphasize the need for standardized acquisition protocols, predefined quality criteria, and explicit reporting of probe type in future elastography studies.

In conclusion, the proof-of-concept data presented here support the hypothesis that TE is a feasible method for conducting early, non-invasive hemodynamic surveillance following EVL. The data provide a rationale for larger, adequately powered studies incorporating serial time points, direct portal pressure assessment where feasible (e.g., HVPG), and clinically relevant endpoints such as rebleeding and decompensation. Future research should include the following elements: firstly, establishing standardized probe selection and quality metrics; secondly, integrating SS into monitoring algorithms; and thirdly, establishing actionable elastography thresholds that can be embedded within contemporary non-invasive strategies for managing PH.

The following limitations must be noted:

- The limited sample size, particularly in subgroup analyses, reduces statistical power and limits generalizability.
- It is hypothesized that cohort heterogeneity, comprising factors such as disease stage, prior ligation history, TIPS/PVT, and probe type, has increased variability in elastography readouts.
- Follow-up was restricted to a single short post-EVL time point (approximately 12 h), which precluded the assessment of longer-term trajectories and recovery dynamics.
- Despite the existing literature establishing correlations between stiffness and portal pressure in the existing literature, no HVPG measurements were available for direct correlation with elastography findings.
- It is possible that technical factors intrinsic to TE, including 100-Hz SSM variability, XL-probe effects, and operator and acoustic window constraints, influenced the absolute values. However, it is clear that the pre/post trends were preserved.

Despite these limitations, this proof-of-concept study provides preliminary evidence of dynamic SS behavior following EVL, thus supporting the feasibility of early, non-invasive hemodynamic monitoring in PH. Further adequately powered studies with serial time points and pressure validation are therefore necessary.

## 5. Conclusions

This pilot study found that liver and spleen stiffness increased consistently, albeit not significantly, within 12 h of EVL. These changes are most likely indicative of acute hemodynamic alterations, such as increased hepatic perfusion and splenic congestion, rather than structural remodeling. Importantly, the findings support the use of elastography for peri-procedural surveillance rather than for decision-making regarding EVL itself, as dynamic post-interventional stiffness changes were associated with relevant clinical and laboratory parameters, including creatinine and albumin levels.

Spleen stiffness was found to be highly sensitive to early hemodynamic changes, highlighting its potential value as a non-invasive indicator of portal and splanchnic congestion following variceal ligation. Beyond changes in stiffness alone, these observations support the idea that EVL-induced flow redistribution contributes to a wider range of manifestations of PH in the early post-interventional phase. Despite the limitations inherent in the small sample size and short follow-up duration, this proof-of-concept study establishes a basis for future research incorporating serial assessments, direct portal pressure measurements, and clinically relevant endpoints. The aim of this future research is to further define the role of spleen elastography in post-EVL monitoring and personalized management of advanced liver disease following EVL.

## Figures and Tables

**Table 1:** Sociodemographic and disease characteristics.

	Total (*n* = 50)	EVL (*n* = 21)	No EVL (*n* = 29)	*p*-Value
**Sociodemographic characteristics**				
Age (years)	59.10 ± 16.17	58.41 ± 11.53	59.65 ± 18.94	0.104
Female:Male	34:16	14:7	20:9	0.740
**Disease characteristics, complications in the past**				
MELD score	8.95 ± 5.21 ^(*n* = 46)^	8.95 ± 5.21 ^(*n* = 20)^	9.08 ± 1.20 ^(*n* = 26)^	0.749
Ascites (yes:no)	35:14	17:3	18:11	<0.001
Child-Pugh score (A:B)	43:5	22:2	21:3	0.822
Spontaneous bacterial peritonitis (SBP) (yes:no)	48:1	20:0	28:1	0.093
Encephalopathy (yes:no)	45:4	20:0	25:4	<0.001
Hepatorenal syndrome (yes:no)	46:3	19:1	27:2	0.594
Portal vein thrombosis (yes:no)	41:8	16:4	25:4	0.266
**Laboratory characteristics**				
Creatinine (mg/dL)	1.07 ± 0.82	0.92 ± 0.33	1.22 ± 1.10	0.199
Total bilirubin (mg/dL)	2.64 ± 5.59	2.54 ± 5.24	2.73 ± 6.01	0.453
ALT (U/L)	45.85 ± 43.94	48.96 ± 41.71	42.75 ± 46.74	0.315
Hemoglobin (g/dL)	11.82 ± 2.07	12.18 ± 1.92	11.45 ± 2.20	0.227
Platelet count (×10^9^/L)	125.96 ± 88.00	99.29 ± 43.37	153.78 ± 112.43	0.050
INR	1.23 ± 0.20	1.20 ± 0.16	1.25 ± 0.24	0.361
**Indication for ligation**				
Primary prophylaxis	12 (24%)	12 (57%)	0 (0%)	<0.001
Secondary prophylaxis	38 (76%)	9 (43%)	29 (100%)	<0.001

## Data Availability

The data presented in this study are not publicly available due to privacy and ethical restrictions. Data may be made available from the corresponding author upon reasonable request and subject to institutional approval.

## Supplementary Materials

The following supporting information can be downloaded at: https://www.mdpi.com/article/10.3390/jcm15020816/s1, Figure S1: The association between changes in liver and spleen stiffness, and the correlation between changes in liver stiffness and baseline spleen stiffness. Figure S2: Shows individual pre- and post-ligation liver and spleen stiffness measurements, stratified by Child–Pugh score.

## Abbreviations

The following abbreviations are used in this manuscript:

ACLD	Advanced Chronic Liver Disease
EGD	Esophagogastroduodenoscopy
EVL	Endoscopic Variceal Ligation
HRVs	High-Risk Varices
HVPG	Hepatic Venous Pressure Gradient
kPa	Kilopascal
LS	Liver Stiffness
LSM	Liver Stiffness Measurement
MELD	Model for End-Stage Liver Disease
PH	Portal Hypertension
pSWE/2D-SWE/TE	Point/2-D Shear Wave Elastography/Transient Elastography
SS	Spleen Stiffness
SSM	Spleen Stiffness Measurement
TE	Transient Elastography
TIPS	Transjugular Intrahepatic Portosystemic Shunt
XL	Extra-Large Probe
