# Supplementary material for: Acute Changes in Liver and Spleen Stiffness Following Endoscopic Variceal Ligation in Advanced Liver Disease—A Pilot Study
Source: J Clin Med. 2026 Jan 20;15(2):816. doi: 10.3390/jcm15020816 (PMC12841821; doi:10.3390/jcm15020816)
Supplement: Supplementary file 1 [file jcm-15-00816-s001.zip › jcm-4078195-supplementary.pptx]

## Slide 1
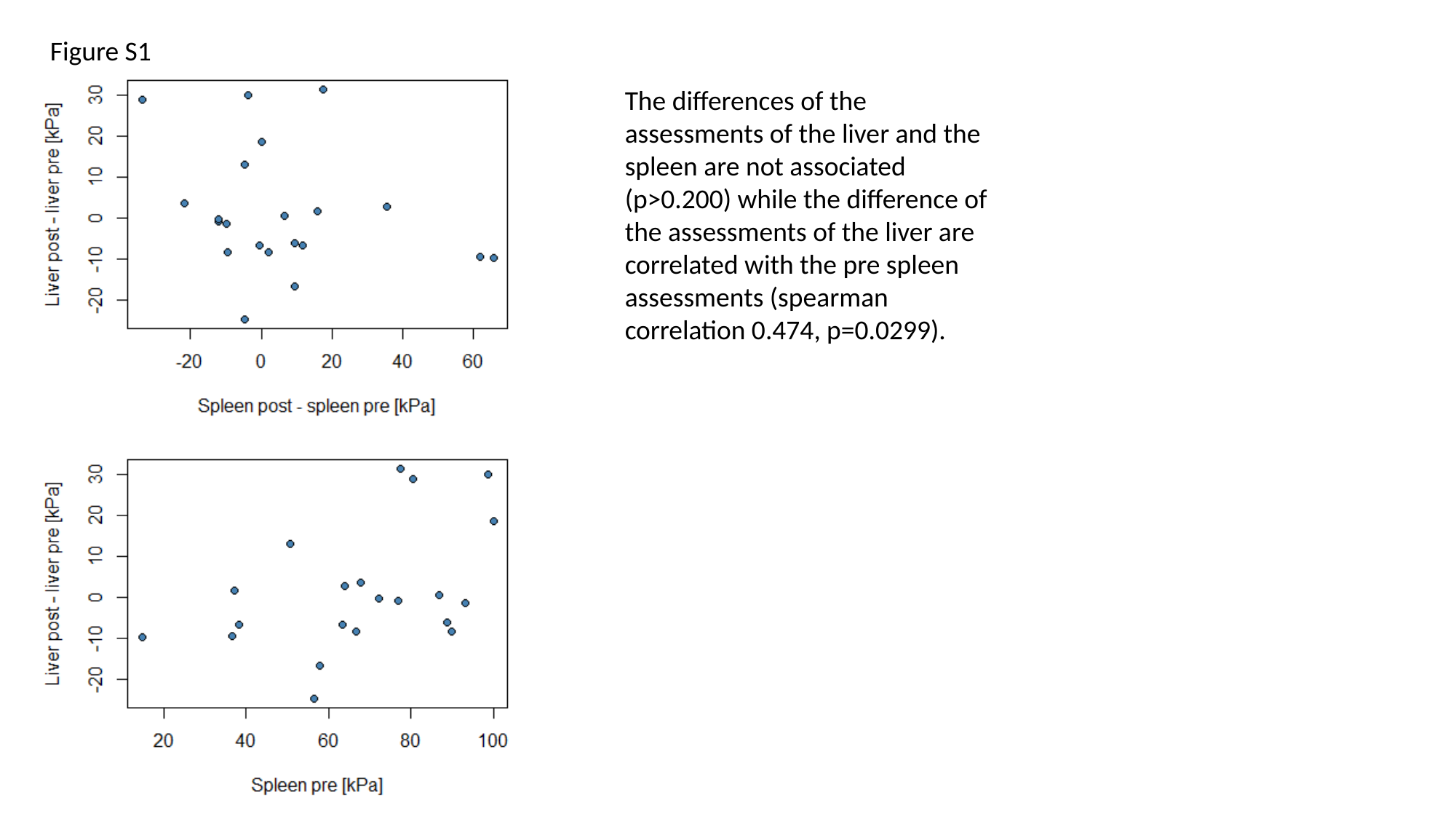

Figure S1
The differences of the assessments of the liver and the spleen are not associated (p>0.200) while the difference of the assessments of the liver are correlated with the pre spleen assessments (spearman correlation 0.474, p=0.0299).

## Slide 2
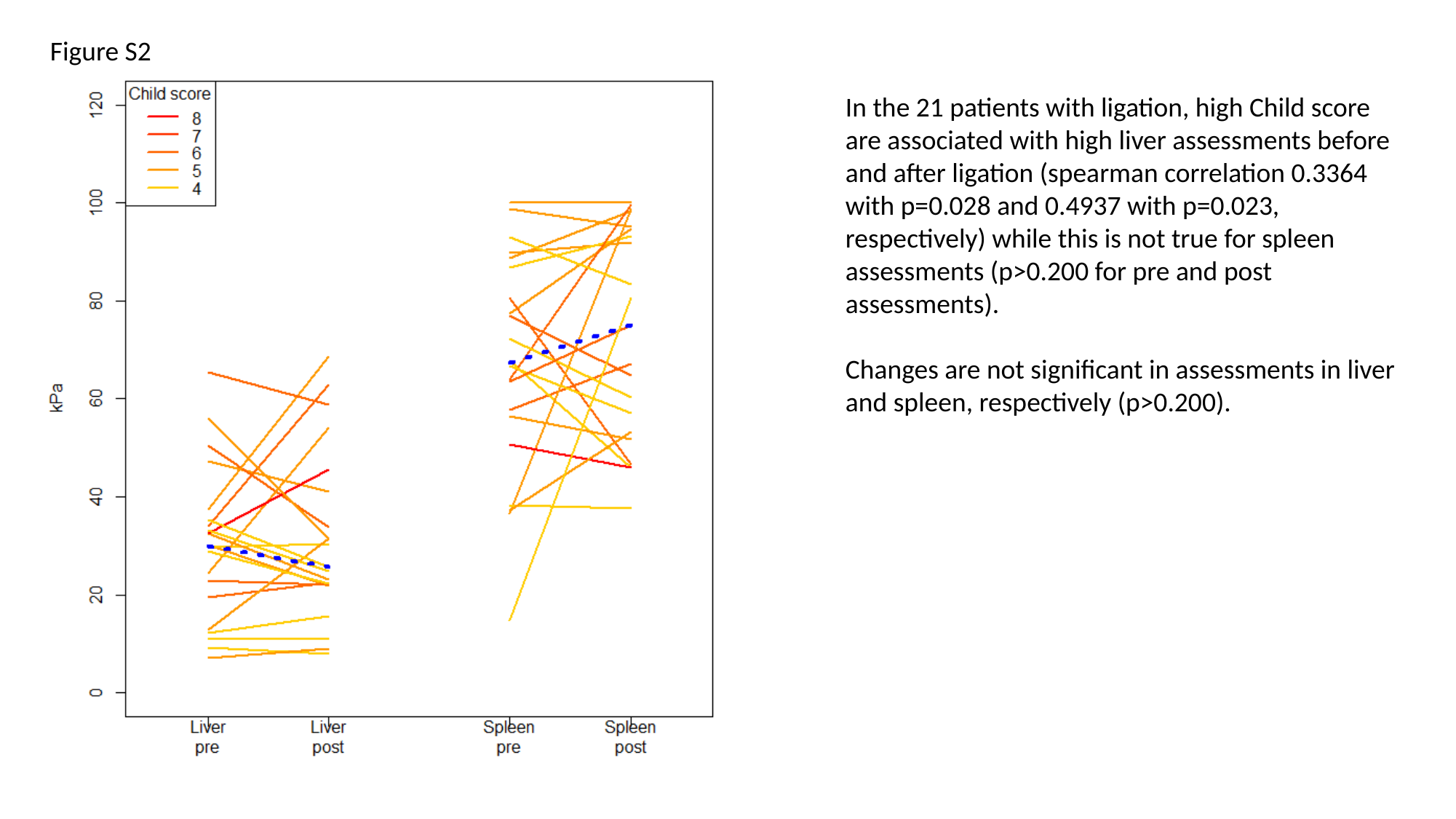

Figure S2
In the 21 patients with ligation, high Child score are associated with high liver assessments before and after ligation (spearman correlation 0.3364 with p=0.028 and 0.4937 with p=0.023, respectively) while this is not true for spleen assessments (p>0.200 for pre and post assessments).
Changes are not significant in assessments in liver and spleen, respectively (p>0.200).
